# Supplementary material for: Substrate Specificity of Biofilms Proximate to Historic Shipwrecks
Source: Microorganisms. 2023 Sep 27;11(10):2416. doi: 10.3390/microorganisms11102416 (PMC10608953; doi:10.3390/microorganisms11102416)
Supplement: Supplementary file 1 [file microorganisms-11-02416-s001.zip › Mugge_MDPImicroSI_figures_supp_info.pdf]

Supplementary figures

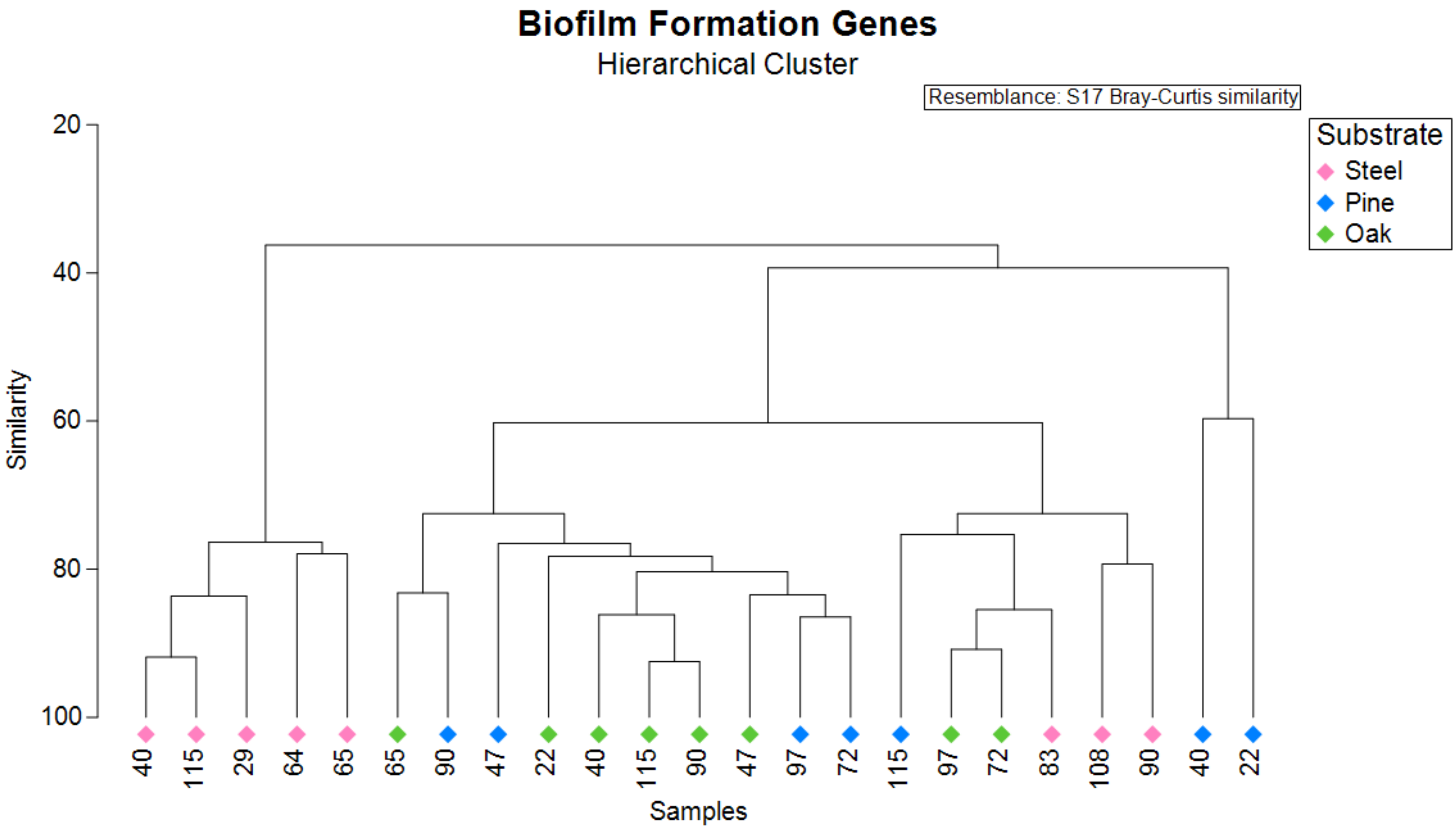

Figure S1. Hierarchical cluster dendrogram based on a Bray-Curtis dissimilarity matrix of normalized gene abundance for biofilm formation genes. Icons are colored by substrate and annotated with distance from structure in meters.

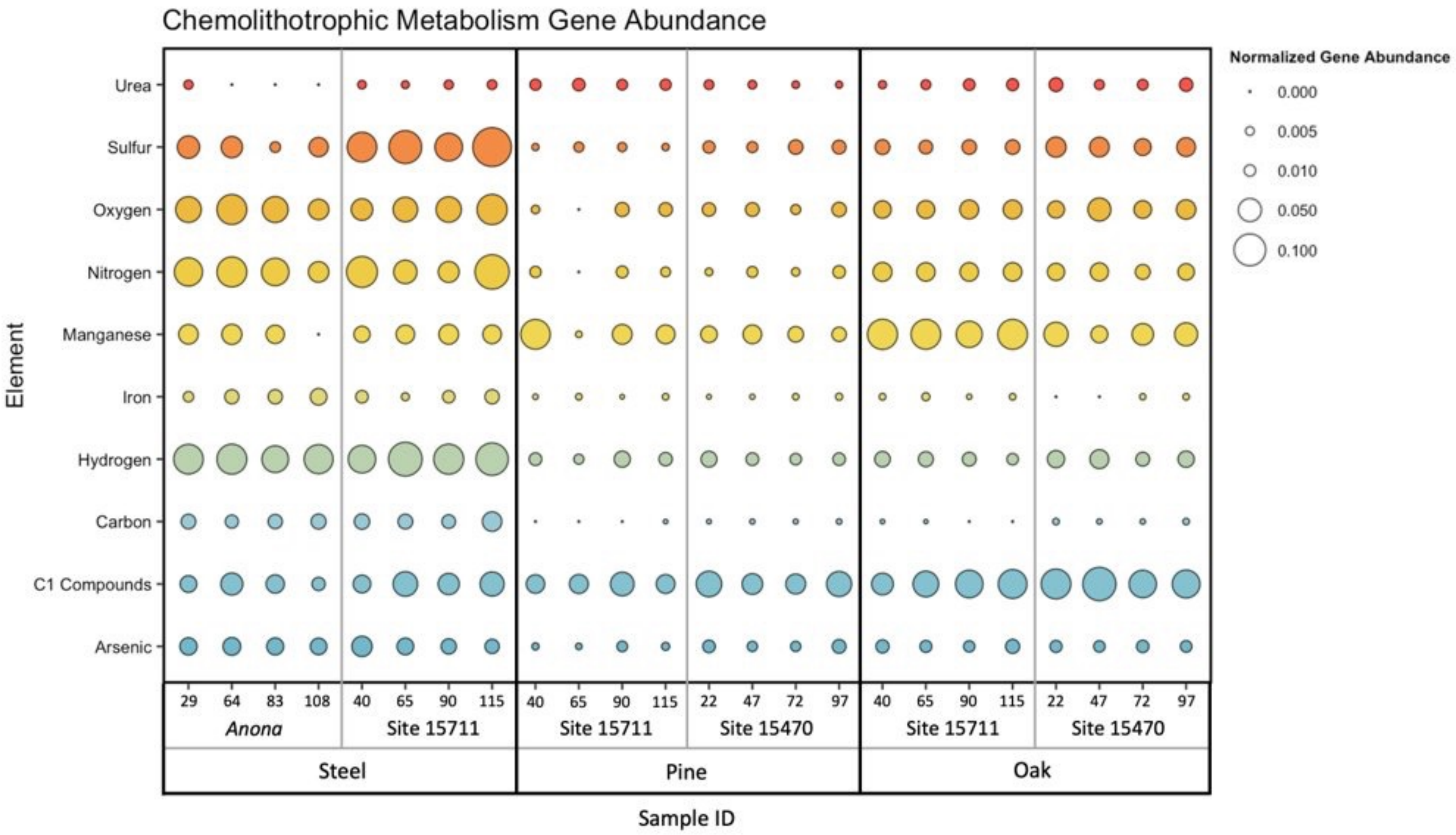

Figure S2. Bubble plot of normalized gene abundance of microbial chemolithotrophic metabolisms within metagenomes. Genes were classified to elements (color) prior to constructing plot. Size represents normalized abundance. Samples are arranged on the X-axis primarily by substrate, then by site, then by distance from structure in meters.
